# Analysis of Survival Modification by Furosemide Use in a Cohort of Hospitalized COVID-19 Patients with Severe or Critical Disease in Mexico: Due to Its Chemical Structure, Furosemide Is More than Just a Diuretic

**DOI:** 10.3390/pharmaceutics16070920

**Published:** 2024-07-10

**Authors:** Janet Diaz-Martinez, Wayne Kotzker, Martha A. Mendoza-Hernandez, Rajdeep S. Gadh, Gustavo A. Hernandez-Fuentes, Andrew Bañuelos, José Guzmán-Esquivel, Angelina Hong, Osiris G. Delgado-Enciso, Elizabeth Geyer-Roberts, Margarita L. Martinez-Fierro, Iram P. Rodriguez-Sanchez, Idalia Garza-Veloz, Luis M. Canseco-Ávila, Ivan Delgado-Enciso

**Affiliations:** 1 Research Center in Minority Institutions, Robert Stempel College of Public Health, Florida International University, Miami, FL 33199, USA; jdimarti@fiu.edu; 2 Florida Kidney Physicians, Panoramic Health Practice, Boca Raton, FL 33431, USA; wkotzker@flkidney.com; 3 Department of Molecular Medicine, School of Medicine, University of Colima, Colima 28040, Mexico; mendoza_martha@ucol.mx (M.A.M.-H.); gahfuentes@gmail.com (G.A.H.-F.); 1933osiris@gmail.com (O.G.D.-E.); 4 COVID Unit, General Hospital Number 1, Mexican Institute of Social Security, Villa de Alvarez, Colima 29883, Mexico; 5 Florida Kidney Physicians, Panoramic Health Practice, Coral Springs, FL 33071, USA; rgadh@flkidney.com; 6 Department GME (General Medicine Education), Hospital Corporation of America Westside, Westside, FL 33324, USA; andrew.banuelos@hcahealthcare.com (A.B.); angelina.hong@hcahealthcare.com (A.H.); 7 Clinical Epidemiology Research Unit, Mexican Institute of Social Security, Villa de Alvarez, Colima 29883, Mexico; jose.esquivel@imss.gob.mx; 8 Department of Medicine, Dr. Kiran C. Patel College of Osteopathic Medicine, Nova University, Fort Lauderdale, FL 33328, USA; egeyerroberts@gmail.com; 9 Molecular Medicine Laboratory, Academic Unit of Human Medicine and Health Sciences, Autonomous University of Zacatecas, Zacatecas 98160, Mexico; margaritamf@uaz.edu.mx (M.L.M.-F.); idgarve@gmail.com (I.G.-V.); 10 Molecular and Structural Physiology Laboratory, School of Biological Sciences, Autonomous University of Nuevo Leon, San Nicolas de los Garza 66455, Mexico; iramrodriguez@gmail.com; 11 Diagnostic and Molecular Biomedicine Laboratory, Faculty of Chemistry Sciences, Campus IV, Autonomous University of Chiapas, Tapachula 30700, Mexico; cansecoavila@gmail.com; 12 Department of Research, Colima Cancerology State Institute, Mexican Institute of Social Security (IMSS-Bienestar) Colima, Colima 28085, Mexico; 13 Department of Dietetics and Nutrition, Robert Stempel College of Public Health and Social Work, Florida International University, Miami, FL 33199, USA

**Keywords:** COVID-19, furosemide, hospital mortality, cohort, survival, structure–activity relation

## Abstract

In the ongoing fight against Coronavirus Disease 2019 (COVID-19), researchers are exploring potential treatments to improve outcomes, especially in severe cases. This includes investigating the repurposing of existing medications, such as furosemide, which is widely available. This study aimed to evaluate the impact of furosemide on mortality rates among COVID-19 patients with severe or critical illness. We assessed a cohort of 515 hospitalized adults who experienced a high mortality rate of 43.9%. Using a multivariate analysis with adjusted risk ratios (AdRRs), factors like smoking (AdRR 2.48, 95% CI 1.53–4.01, *p* < 0.001), a high Pneumonia Severity Index (PSI) score (AdRR 7.89, 95% CI 5.82–10.70, *p* < 0.001), mechanical ventilation (AdRR 23.12, 95% CI 17.28–30.92, *p* < 0.001), neutrophilia (AdRR 2.12, 95% CI 1.52–2.95, *p* < 0.001), and an elevated neutrophil-to-lymphocyte ratio (NLR) (AdRR 2.39, 95% CI 1.72–3.32, *p* < 0.001) were found to increase mortality risk. In contrast, vaccination and furosemide use were associated with reduced mortality risk (AdRR 0.58, *p* = 0.001 and 0.60, *p* = 0.008; respectively). Furosemide showed a pronounced survival benefit in patients with less severe disease (PSI < 120) and those not on hemodialysis, with mortality rates significantly lower in furosemide users (3.7% vs. 25.7%). A Kaplan–Meier analysis confirmed longer survival and better oxygenation levels in patients treated with furosemide. Furthermore, a Structure–Activity Relationship analysis revealed that furosemide’s sulfonamide groups may interact with cytokine sites such as tumor necrosis factor-alpha (TNF-α) and interleukin-6 (IL-6), potentially explaining its beneficial effects in COVID-19 management. These findings suggest that furosemide could be a beneficial treatment option in certain COVID-19 patient groups, enhancing survival and improving oxygenation.

## 1. Introduction

The emergence of the novel severe acute respiratory syndrome coronavirus 2 (SARS-CoV-2) has led to the global pandemic of Coronavirus Disease 2019 (COVID-19), characterized by a spectrum of symptoms ranging from mild respiratory distress to severe pneumonia, often culminating in multiple organ failure and death [1]. As of March 2024, the World Health Organization (WHO) has reported an alarming toll of more than 7.04 million deaths worldwide [2], while in Mexico alone, 335 thousand COVID-19-related deaths have been documented [3].

In response to this unprecedented health crisis, significant strides have been made in the development of vaccines and innovative therapies to treat the disease, including anti-virals, anti-inflammatories, respiratory therapy, and antibody therapies, which are essential components of SARS-CoV-2 infection treatment [4]. However, the widespread availability of these therapies remains a challenge, particularly in resource-limited settings and among marginalized socioeconomic groups [5]. Therefore, investigating more accessible therapeutic options becomes imperative to ensure equitable access to essential medical interventions. In this regard, studies exploring the use of other therapies including furosemide have gained importance, as it holds promise in addressing some of these pressing needs, both in affluent nations and resource-constrained regions [5,6,7].

Many drugs, originally targeting conditions other than COVID-19, have shown effects on disease complications, primarily acting on cytokine-related inflammatory pathways or intervening via other mechanisms (promoting antioxidant activity and cyclooxygenase inhibition). Some of these drugs share similar mechanisms of action and are currently under investigation in clinical trials. Some examples of these repurposed drugs that are being tested are esomeprazole, fentanyl, fluticasone, calcium gluconate, guaifenesin/dextromethorphan, heparin, indomethacin, linezolid, digoxin, isosorbide dinitrate, diosmectite, torasemide, piretanide, bumetanide, ebselen, some montelukast derivatives, and furosemide, among others [8,9,10]. These drugs were integrated into protocols to be used as a strategy for moderate to severe cases of COVID-19 [8]. 

One of these repurposed drugs is furosemide. In Mexico, furosemide was administered to patients requiring hospitalization and intensive therapy based on the recommendations outlined in the “Proposal of Medications for the Treatment of COVID-19” (Propuesta de medicamentos para el tratamiento de COVID-19, in Spanish), published by the Undersecretariat of Health Sector Integration and Development (Subsecretaría de Integración y Desarrollo del Sector Salud, in Spanish) in collaboration with the Ministry of Health (Secretaría de Salud, in Spanish) [8]. However, furosemide was not administered as primary therapy for COVID-19 but rather as part of an optimal treatment approach based on the baseline and comorbid conditions of the patients. This included metabolic control, antihypertensive control, renal replacement therapy, antibiotic therapy, high-flow oxygen support both invasive and non-invasive, the treatment of renal injury, edema and fluid control, and anti-inflammatory therapy, among others, during their hospitalization. The treatment was tailored to the patient’s evolution under the guidance of specialist physicians. Such therapeutic guidelines emerged in various countries in the early years of the pandemic from 2020 to 2022, during which new knowledge and pharmaceutical developments were generated that have led to the current guidelines [11].

The potential utility of furosemide in the context of COVID-19 infection, which is a known loop diuretic [12], lies in its ability to be repurposed and potentially utilized for treating conditions beyond its primary indication [7]—such as hypertension and heart failure—and it is available in reasonable quantities worldwide [6,7,13]. It has been postulated that furosemide can address the cytokine storm associated with COVID-19 [1,6,13] due to its ability to inhibit cytokine production. Cytokines are pivotal signaling molecules in the immune response [14,15]. Therefore, it is hypothesized that furosemide could improve outcomes among COVID-19 patients, especially considering the link between severe disease and elevated inflammatory markers [16]. However, investigations into its impact on mortality in COVID-19 patients have yielded conflicting results [17,18,19,20,21].

This study aims to identify factors associated with mortality in hospitalized COVID-19 patients with severe or critical disease, focusing specifically on the impact of furosemide use on survival outcomes. By understanding the role of furosemide alongside other clinical factors, we seek to elucidate its efficacy as a supportive treatment in managing severe COVID-19 infections. Additionally, the study incorporates an analysis of the structural characteristics of furosemide, comparing them with other molecules used in the treatment of COVID-19. This comparison has revealed certain structural features that may open the door to new research avenues, potentially leading to significant advancements in the treatment of severe COVID-19 or similar illnesses.

## 2. Materials and Methods

### 2.1. Study Subjects

**Figure 1:**
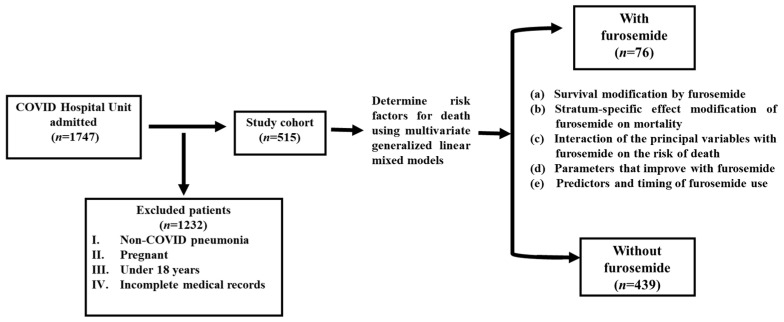
Selection process from a total of 1747 hospitalized individuals based on predefined inclusion and exclusion criteria.

The study was a single-center ambispective (bidirectional) cohort study conducted at General Hospital Number 1 of Mexican Institute of Social Security (IMSS)-Colima, Mexico, focusing on patients admitted to the COVID-19 unit between February 2021 and December 2022 with severe or critical disease. Information was collected from patients who were receiving furosemide treatment and those who were not. It is important to note that this was an observational cohort study, analyzing clinical and therapeutic data from the routine medical management provided by the treating specialists. The patients received an appropriate clinical and therapeutic approach based on the clinical evolution of each patient, largely based on the Mexican guidelines that emerged during the development of the study [22]. The development of this study did not intervene in any aspect of patient care. The research protocol received approval from the local health research committee of IMSS-Colima, Mexico (approval R-2021-601-014, dated 30 June 2020). Since the data were sourced from clinical records, the ethics committee exempted the need for signed consent from individual subjects (based on regulations of the General Health Law on Health Research, Mexico) [23]. This is because all diagnostic and therapeutic procedures were part of the usual medical management, and the research protocol was observational. It did not represent any additional risk to the patients. Anonymity was preserved, and personal identification details were masked in the databases used for the analysis. The study was reported according to the Strengthening the Reporting of Observational Studies in Epidemiology (STROBE) Statement guidelines for reporting observational studies [24]. The study included men and nonpregnant women aged 18 or older who tested positive for COVID-19 through SARS-CoV-2 RT-PCR or antigen tests. Patients admitted to regular hospital floors, high-flow oxygen rooms, or ICUs were eligible, while those treated solely in the emergency room were excluded. After screening 1747 hospitalized individuals, 515 met the predetermined inclusion and exclusion criteria (see Figure 1) after excluding subjects with missing vaccination information or incomplete clinical records.

### 2.2. Measures and Follow-Up

Patient data from clinical records encompassed personal history, COVID-19 vaccination status, and various clinical parameters monitored during hospitalization until discharge, whether due to recovery or death. Key variables included demographics; medical history (including comorbidities and Charlson comorbidity index scores) [25]; specifics of COVID-19 vaccination; history of prior COVID-19 infections; smoking status (limited to current smokers) [26]; admission disease phase (viral/pulmonary/hyperinflammatory); daily clinical, laboratory, and imaging findings; and discharge outcomes. 

Repeatedly documented data during hospitalization included blood cell counts, inflammation markers, glucose and creatinine levels, liver enzymes, prothrombin time, mechanical ventilation or hemodialysis usage, medication administration, and risk score calculations using various clinical parameters like Pneumonia Severity Index (PSI), National Early Warning Score 2 (NEWS-2), and COVID-GRAM [27]. The glomerular filtration rate was estimated from serum creatinine using the CKD-EPI 2021 equation. These data were recurrently recorded throughout the entire hospital stay. Neutrophilia, indicative of possible bacterial coinfection, was considered a neutrophil count equal to or greater than 9100 cells/μL, a figure previously reported for patients hospitalized for COVID-19 with confirmed bacterial infection [28].

### 2.3. Sample Size

Previously, a clinical trial was reported in intubated patients with COVID-19, evaluating treatment with nebulized furosemide versus placebo. However, it did not yield significant results due to premature termination, resulting in inadequate statistical power. Nonetheless, a 60-day mortality rate of 48% versus 71% was reported in the furosemide and placebo groups, respectively [29]. Based on these findings, our study calculated a sample size of 70 patients in each group (furosemide and non-furosemide) to achieve a desired power of 80%. Subsequently, a post hoc statistical power analysis was conducted after the study’s completion, demonstrating that systemic furosemide administration, in the stratum of less severe patients (PSI < 120) without hemodialysis, reduces mortality in hospitalized COVID-19 patients, resulting in a statistical power of 87.4% (α = 0.05).

### 2.4. Statistical Analysis

The normality of data was assessed with Kolmogorov–Smirnov tests. Data representation utilized percentages or averages and standard deviation. Quantitative data with non-normal distribution are expressed as medians and 25–75th percentiles (Q1–Q3). To compare three groups (intergroup analysis), an ANOVA or a Likelihood ratio chi-square test was performed, and to compare two groups, an independent Student’s *t*-test or Fisher’s exact test was used, as appropriate for numerical or qualitative data, respectively. An association analysis was performed utilizing multivariate generalized linear mixed models (GLMM, GENLINMIXED in SPSS) employing a binary logistic regression link and distinct random intercepts (SPRI), following established methodology [27,30]. To accommodate the longitudinal aspect of the data, two random variables were included: (I) hospitalization day and (II) pandemic periods. Periods were established based on the predominant variants in the general population of Mexico (January–July 2021: B.1.1.222; B.1.1.519; and Gamma. August–December 2021: Delta. January–April 2022: Delta, Omicron BA.1; Omicron BA.1.1, Omicron BA.1.15. May–December 2022: Omicron, others) [27,30,31]. The outcome variable assessed was patient mortality during hospitalization (dichotomous; yes or no); fixed effects comprised dichotomous variables representing different clinical characteristics and vaccine categories. Numeric clinical variables were dichotomized using cutoff points derived from receiver operating characteristic (ROC) curve analyses predicting patient mortality. Covariance structures were determined using Akaike’s Information Criterion (AIC). The primary aim of the model was to derive marginal risk estimates by aggregating binomial regression parameters into adjusted relative risk (AdRR) values with corresponding 95% confidence intervals (CI) and *p*-values [32]. Two models were generated to determine the RR for death of various variables: (A) Model 1 included the variables that were different (*p* < 0.05) between patients who received or did not receive furosemide during their hospitalization, adding the variables of mechanical ventilation and smoking, because they are considered relevant predictors of death, and (B) Model 2, which only included the variables that resulted with a *p* < 0.150 in the analysis of Model 1. Additionally, stratum-specific furosemide effect modification on the risk of death was analyzed. To this end, the effect of furosemide on the risk of death was analyzed in different strata, with the absence or presence of each clinical variable included in model 2. The above was conducted to identify relevant variables to perform an interaction analysis (involving a two- or three-way interaction). The values of various clinical characteristics throughout the entire hospitalization period were analyzed to determine if they were able to predict the use of furosemide in patients hospitalized for COVID-19, using the area under the ROC curve (AUC), the confidence interval, and the cutoff point. This analysis was performed separately for the stratum of patients who survived and those who died, and it was also used to analyze whether there were differences between the AUC of the two mentioned strata. The survival probabilities were estimated using a Kaplan–Meier analysis (log-rank test). Statistical power and sample size were calculated using ClinCalc version 1 [33,34,35]. All other analyses were performed using SPSS Statistics version 20 software (IBM et al., USA). A significance level of *p* < 0.05 was considered statistically significant.

### 2.5. Structure–Activity Analysis

The Structure–Activity Relationship (SAR) analysis was performed using a comprehensive bibliographic review that highlighted the pharmacophores responsible for the biological activity of compounds within the furosemide family, including torasemide, piretanide, and bumetanide, as well as molecules related to montelukast and ebselen [9,36,37]. Molecular illustrations were created using ChemDraw 3D, version 20.0 software (PerkinElmer, USA) [38], where each molecule was structurally optimized and key properties such as ClogP were calculated.

## 3. Results

### 3.1. Patient Characteristics and Generalities

Five hundred and fifteen hospitalized patients were enrolled in the study. Among them, 439 (85.2%) had not received furosemide during hospitalization, while 76 (14.8%) had received it at some point during their hospital stay. These patients collectively accounted for a total of 4944 days of hospitalization. The length of hospital stay exhibited a non-normal distribution; therefore, it is best to use the median. Thus, the median hospital stay (Q1–Q3) was 7.0 (4–11) days, and there was a high mortality rate of 43.9%.

Table 1 presents the main clinical characteristics of the participants at the time of enrollment and the prescribed treatments during hospitalization, categorized by whether they received furosemide. Overall, it can be observed that compared to those who did not receive furosemide, patients who did receive it had more adverse prognostic factors upon hospital admission, including higher comorbidities (Charlson Index 3.5 ± 2.1 vs. 4.5 ± 1.8, *p* < 0.001), greater severity (PSI 104 ± 41 vs. 117 ± 28, *p* = 0.048), increased inflammation (higher levels of NLR and ferritin), evidence of bacterial coinfection (neutrophilia), and significantly lower renal function (eGFR) upon admission (72.0 ± 46.4 vs. 30.2 ± 27.2 mL/min/1.73 m^2^, *p* < 0.001). This is consistent with the fact that patients who received furosemide also received other treatments in higher proportions, such as antibiotics (46.3% vs. 58.6%, *p* = 0.038) or hemodialysis (6.0% vs. 34.7%, *p* < 0.001). The only favorable aspect in the subgroup that received furosemide was a higher percentage of vaccinated individuals, with at least one dose (41.5% vs. 55.6%, *p* = 0.029) or a complete initial vaccination scheme (35.1% vs. 50.8%, *p* = 0.019). Only two patients reported having had a previous COVID-19 infection, so this characteristic was not included as a variable in the analyses.

Table 2 presents a multivariate analysis that identifies various factors associated with death in hospitalized patients with COVID-19, as well as the impact of furosemide use on mortality. The analysis encompassed data from the entire hospitalization period of patients (repeated measurements using mixed models) rather than solely admission data. Two statistical models were employed: (A) Model 1 comprised variables that exhibited differences between groups, as shown in Table 1, along with mechanical ventilation and smoking status, which were deemed relevant predictors of death, and (B) Model 2, which only comprised variables with a significance level of *p* < 0.150 from Model 1. 

Model 2 (see Table 2)—the final statistical model that considered pre-existing conditions or conditions upon hospital admission—showed that smoking (RR 2.5, 95% CI 1.5–4.0, *p* < 0.001) and advanced disease phase (pulmonary/hyperinflammatory) significantly increased the risk of death (RR 1.9, 95% CI 1.4–2.6, *p* < 0.001). In the same Model 2 (Table 2), it can be observed that during hospitalization, an elevated PSI score >120 (RR 7.9, 95% CI 5.8–10.7, *p* < 0.001), the need for mechanical ventilation (RR 23.1, 95% CI 17.3–30.9, *p* < 0.001), neutrophilia > 9.1 × 10^3^/µL (RR 2.1, 95% CI 1.5–2.9, *p* < 0.001), and an elevated NLR (RR 2.4, 95% CI 1.7–3.3, *p* < 0.001) were identified as risk factors. Conversely, being vaccinated (RR 0.58, 95% CI 0.4–0.8, *p* = 0.001), furosemide use (RR 0.60, 95% CI 0.4–0.8, *p* = 0.008), and having ALT > 45 UL (RR 0.6, 95% CI 0.5–1.0, *p* = 0.049) were identified as protective factors.

### 3.2. Our Approach to Analyzing the Stratum-Specific Effect Modification of Furosemide on Mortality

In our pursuit of detecting interactions between variables, we explored various methods, ultimately identifying the stratified-specific approach as the most suitable for our initial data analysis. Effect modification, which involves stratification, occurs when an exposure produces varying effects across different subgroups. Effect modification is associated with the outcome but not directly with the exposure. This method entails visually inspecting the data to identify patterns of variation in the stratum-specific estimates. Consistency across these estimates suggests no interaction, while differences indicate its presence, thereby prompting a focus on describing how the association of interest is influenced by the stratifying factor. However, determining an interaction can be challenging due to potential random variation in stratum-specific estimates [34,35]. Although various χ^2^ tests of heterogeneity can assess this variability, they may lack power, especially in epidemiological studies with limited statistical capacity [34,35]. It is important to note that although stratum-specific effect modification has been used to search for and suggest possible interactions, effect modification can be present with no interaction and interaction can be present with no effect modification [39]. 

Our analysis delved into understanding how the impact of furosemide on mortality varies based on specific patient characteristics or conditions. We conducted a stratified analysis, dividing the study population into subgroups based on significant clinical variables identified in the previous multivariate analysis (Table 2, Model 2). This approach allowed us to examine the effect modification of furosemide on mortality, providing insights into its relationship with fatal outcomes in COVID-19 patients. By analyzing each stratum separately, we aimed to elucidate how furosemide’s effect may differ across diverse patient profiles while mitigating potential confounding variables within each stratum by using a multivariate model for each stratum [40,41,42,43] (see Table 3).

Table 3 presents the results of furosemide’s effect on fatal outcomes in different strata, according to the presence or absence of a clinical characteristic, in patients hospitalized for COVID-19. A visual inspection of the results to identify patterns or variations across different strata—an approach that has been reported previously [42,44,45,46]—revealed variations in the effect of furosemide on the specific relative risk (AdRR) depending on the analyzed stratum (see Table 3). For instance, the protective effect of furosemide against mortality was more pronounced in the absence of adverse factors, such as non-smoking status, the absence of hemodialysis requirement, the absence of suspected bacterial infection (no neutrophilia or antibiotic use), a PSI score <120, and lower inflammation, indicated by NLR (see Table 3). On the other hand, in the strata of patients that exhibited adverse prognostic factors, the protective effect of furosemide was more prominent in non-vaccinated patients (adjusted RR = 0.53; 95% CI 0.36–0.78) than in vaccinated patients (adjusted RR = 0.87, 95% CI 0.39–1.90), suggesting a weak modification effect of vaccination status over furosemide protection (see Table 3). However, this difference in RRs among strata may reflect a random variation, which could also occur in other variables where the effect of furosemide in both strata was small or in the same direction [47].

Overall, the results from Table 3 show that while the modification of furosemide’s effect on the risk of death was not substantial across most patient strata, a significant change in the effect of furosemide on the risk of death was evident in the group of patients with or without a PSI score >120 (adjusted RR = 1.76, 95% CI 0.96–3.24; adjusted RR = 0.25, 95% CI 0.14–0.46, respectively) and hemodialysis (adjusted RR = 2.30, 95% CI 0.73–7.23; adjusted RR = 0.45, 95% CI 0.30–0.68, respectively). Therefore, these variables were further included in a model that incorporated three-way interactions (furosemide use *PSI score >120 *hemodialysis), along with adjustment variables of mechanical ventilation, a Charlson index >8, and vaccination status, based on the most relevant predictors of death identified in Table 2. 

Table 4 displays the results of this interaction analysis, reaffirming that the protective effect of furosemide is mainly observed in patients with lower severity (PSI score <120 and no hemodialysis requirement). In these patients, furosemide administration reduced the probability of death fourfold (adjusted RR = 0.23; 95% CI 0.15–0.34). Conversely, in severe patients with a PSI score >120, the risk of death was not modified by furosemide administration, and in patients requiring hemodialysis, furosemide use was associated with an increased risk of death (adjusted RR = 15.99, 95% CI 9.77–26.18; adjusted RR = 5.74, 95% CI 4.02–8.20, with and without furosemide, respectively) (see Table 4). Table 5 presents the mortality of patients in the stratum of less severe patients (PSI < 120) without hemodialysis, indicating mortality rates of 3.7% and 25.7% among patients with and without furosemide use, respectively (*p* = 0.008). This analysis achieved a post hoc statistical power (α = 0.05) of 87.4%.

### 3.3. Analysis of Survival Modification by Furosemide

**Figure 2:**
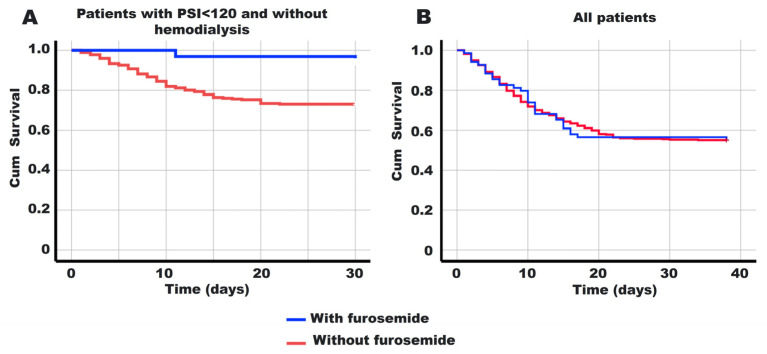
Kaplan–Meier curves for 40 days based on furosemide treatment. (**A**) Individuals receiving furosemide, with a PSI score <120 and without hemodialysis treatment, show a significant increase in survival time among patients compared to those not receiving the drug (*p* = 0.013). (**B**) Among all hospitalized patients, there was no significant difference in survival rates between those with and without furosemide (*p* = 0.994).

The analysis of survival outcomes in patients hospitalized for COVID-19 revealed notable differences associated with furosemide use, particularly in individuals with a PSI score <120 and without hemodialysis treatment. Kaplan–Meier curves demonstrated a significant increase in survival time among patients receiving furosemide compared to those not receiving the drug (*p* = 0.013). Specifically, patients in this subgroup who received furosemide exhibited a mean survival time of 29.4 days (95% CI 27.7–31.0) with a survival rate of 96.3%, whereas those without furosemide had a mean survival time of 24.4 days (95% CI 23.2–25.5) with a survival rate of 74.3% (see Figure 2A). However, when considering all hospitalized patients without stratification, there was no significant difference in survival rates between those with and without furosemide (*p* = 0.994, see Figure 2B), with mean survival times of 25.3 days (95% CI 21.8–28.9, survivors 54.9%) and 25.4 days (95% CI 23.9–26.7, survivors 55.1%), respectively. These findings suggest a potential survival benefit associated with furosemide use in specific patient subgroups, warranting further investigation into its therapeutic role in COVID-19 management. Additionally, the length of hospital stays (excluding outcome consideration) for patients with a PSI score <120 and without hemodialysis treatment was 8.0 (Q1–Q3: 5.0–11.5) days with furosemide compared to 6.0 (Q1–Q3: 4.0–9.0) days without (*p* = 0.006), while for the entire cohort, it was 8.0 (Q1–Q3: 5.0–13.0) days with furosemide versus 7.0 (Q1–Q3: 4.0–11.0) days without (*p* = 0.003). 

### 3.4. Oximetry, One of the Parameters That Improves with Furosemide

Considering only the patient strata where furosemide was found to increase survival, it becomes apparent that patients receiving furosemide during their hospital stay present more adverse clinical characteristics upon admission (see Table 6). They exhibit a higher proportion of CKD history (8.3% vs. 40.7%, *p* < 0.001), diminished renal function (84.7 ± 45.9 vs. 37.7 ± 28.1, *p* < 0.001), and overall increased severity (PSI: 81.5 ± 24.3 vs. 95.5 ± 16.2, *p* = 0.004, without vs. with furosemide, respectively). Clinical parameters improved in both groups throughout hospitalization, although patients receiving furosemide maintained more adverse parameters. For instance, the mean PSI was 80.4 ± 24.1 vs. 94.1 ± 16.0 (*p* < 0.001) in patients without and with furosemide, respectively. Mechanical ventilation was required in 11.1% of patients with furosemide, compared to 2.3% without furosemide (*p* = 0.041). While eGFR improved in both groups, it was higher in patients without furosemide (109.6 ± 60.5 vs. 66.0 ± 44.5, *p* < 0.001). Despite more ongoing adverse patient clinical data in the furosemide group, they experienced lower mortality than those who did not receive furosemide (3.7% vs. 25.7%; see Table 5). A close examination of the parameter oximetry showed no significant differences upon admission (92.3 ± 10.5 vs. 93.5 ± 8.9%, *p* = 0.578), but it became significantly higher during hospitalization in patients receiving furosemide—93.5 ± 8.9% (upon admission) and 95.6 ± 3.8% (during hospitalization, *p* = 0.012). 

To confirm the results indicating a higher number of deaths in the non-furosemide group, we reanalyzed certain parameters, considering only patients who survived. It was observed that greater severity persisted in the furosemide group, with a PSI of 76.4 ± 23.6 compared to 93.5 ± 16.4 in patients without and with furosemide, respectively (*p* < 0.001), and higher oximetry levels were maintained (92.8 ± 9.5 vs. 95.6 ± 4.0, *p* = 0.008). It is important to note that in the patients analyzed with these characteristics (those who survived, with PSI < 120 and without the need for hemodialysis), the percentage of fully vaccinated patients did not differ between those who received furosemide and those who did not (86.7% vs. 90.3%, *p* = 0.649, respectively). Therefore, their improvement in oxygenation could not be attributed to vaccination status. Our analysis suggests that oxygenation is one of the clinical parameters through which furosemide might have contributed to increased survival in this subgroup of patients.

### 3.5. Predictors and Timing of Furosemide Use

It is worth noting that in our cohort of hospitalized COVID-19 patients, furosemide administration was primarily based on clinical judgment, often indicated for the clinical management of acute renal failure characterized by elevated serum urea and creatinine levels or fluid accumulation, regardless of renal failure. Table 7 shows the results of the area under the ROC curve (AUC) analysis, indicating that serum urea and creatinine levels, as well as eGFR, were predictive factors for furosemide use in both surviving and deceased patients. However, the cutoff points for furosemide use were lower in surviving patients compared to those who died, suggesting that furosemide was administered during earlier stages of renal impairment in surviving patients. Additionally, the severity of illness (PSI score) predicted furosemide use only in surviving patients, indicating its potential role as a prognostic indicator for furosemide administration. Conversely, oximetry levels did not predict furosemide use. Overall, the findings suggest that the “early” use of furosemide, before significant elevations in urea or creatinine levels, may be associated with improved prognosis in hospitalized patients with COVID-19.

### 3.6. Structure Relationship Analysis

**Figure 3:**
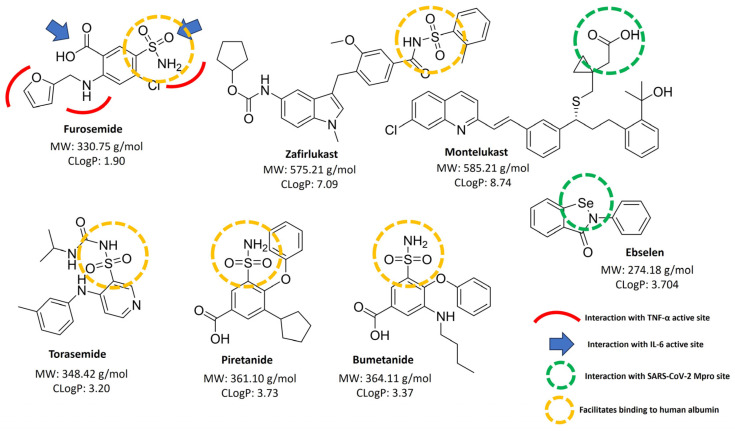
Structural analysis and cytokine interaction of furosemide, ebselen, montelukast, and their analogs.

This analysis was conducted to propose potential activities of furosemide that may contribute to its therapeutic effect in treating COVID-19, in addition to its diuretic effect. Figure 3 illustrates the molecular structures of furosemide compared with drugs from the same chemical family, like torasemide, piretanide, bumetanide, zafirlukast and montelukast that were incorporated, emphasizing key functional groups against COVID-19. These molecules were selected based on their structural similarity to furosemide and comparable chemical characteristics, as well as prior studies where they have been evaluated for their effectiveness against complications arising from COVID-19. Building upon the insights gathered from our Structure–Activity Relationship (SAR) analysis, several significant findings have emerged regarding the therapeutic potential of furosemide in treating severe COVID-19 [37]. These compounds, while sharing similar molecular weights with furosemide, display varying ClogP values. The ClogP, or the partition coefficient, is a measure of a substance’s lipophilicity or hydrophilicity [48]. For instance, the specific ClogP values are 1.90 for furosemide, 3.20 for torasemide, 3.73 for piretanide, and 3.37 for bumetanide.

A compound that has recently garnered interest and been explored in clinical trials for its activity against the SARS-CoV-2 main protease (Mpro) is ebselen [9,10,49], which has a ClogP of 3.704, placing it in a similar range as furosemide and its derivatives. However, the key structural feature for ebselen’s binding to the Mpro enzyme is the linkage between the heteroatom and selenium. While structurally different from furosemide, which uses a sulfonamide linkage crucial for protease binding, ebselen reportedly binds to cysteine residues, facilitating lactam ring disruption and establishing interactions with nucleophiles [49,50,51,52] similar to the previous report to furosemide and some derivatives [53]. Although montelukast and zafirlukast, which are effective leukotriene synthesis inhibitors and interact with SARS-CoV-2 Mpro binding sites, exhibit higher ClogP values of 8.74 and 7.09, respectively, furosemide’s lower ClogP value suggests its suitability for aerosolized treatments, owing to enhanced potential for inhalation delivery. Considering these factors and noting that the optimal ClogP range for therapeutic agents is typically 0 to 5 [48], furosemide’s properties indicate its potential effectiveness in nebulized form for treating COVID-19. However, it is important to note that in this study, furosemide was administered systemically (either orally or intravenously) and not inhaled. It is known that in healthy individuals, over 95% of systemically administered furosemide binds to plasma proteins, predominantly albumin, with only 2.3% to 4.1% remaining unbound in therapeutic concentrations [12]. This high plasma protein binding suggests that furosemide, when administered systemically, remains largely within the circulatory system, potentially enhancing its therapeutic reach and efficacy in targeting tissues affected by COVID-19. This dual potential for systemic and nebulized administration makes furosemide a versatile candidate for further investigation in COVID-19 treatment protocols. On the other hand, montelukast and zafirlukast have different mechanisms—acting as leukotriene synthesis inhibitors—and have demonstrated efficacy in inhibiting COVID-19-related enzymes [36], but their higher molecular weights and ClogP values may limit their effectiveness in aerosol form, supporting furosemide as a potential treatment with a diverse range of administrations. 

The analysis conducted allowed us to identify specific structural features within molecules of the furosemide family that directly interact with key active sites involved in the progression of COVID-19, particularly regarding anti-inflammatory cytokine activity against IL-6 and TNF-α [13]. We observed that the furan ring, amine linkers, and carboxylic acid ends, particularly the sulfonamide group, exhibit the most effective interactions with the active sites of TNF-α [13]. Additionally, both the sulfonamide and carboxyl groups play crucial roles in the inhibition of IL-6 [13].

As shown in Figure 3, torasemide, piretanide, bumetanide, and zafirlukast contain sulfonamides in their structures, which may confer their inhibitory capabilities on TNF-α and IL-6. Furthermore, the presence of sulfonamides is key for binding to human albumin, enhancing their transport in the bloodstream, which could support their clinical use [13]. On the other hand, montelukast has been evaluated for its interaction with the SARS-CoV-2 Mpro site, with less sterically hindered carboxyls being able to engage with the active site effectively [36]. This information could aid in designing molecules that possess these characteristics to achieve more pronounced effects in combating COVID-19.

## 4. Discussion

In our analyzed cohort of hospitalized COVID-19 patients with severe or critical disease, the median hospital stay (Q1–Q3) was 7.0 (4–11) days, with a high mortality rate of 43.9%. This aligns with hospital mortality reports in Mexico, which recorded a rate of 45.1% (95% CI 44.9–45.3) from March 2020 to August 2022, positioning Mexico among the countries with the highest hospital mortality globally during that period [3,54].

Our analysis shows that despite the fact that the COVID-19 patients who received furosemide during their hospitalization tended to have more adverse prognostic factors upon admission (higher burden of comorbidities, greater severity of illness, increased inflammation, bacterial coinfection, and significantly lower renal function), furosemide showed a potential protective effect against mortality, especially on hospitalized patients with less severe disease statuses. In addition, the group of COVID-19 hospitalized patients who received furosemide were more likely to undergo other medical interventions, such as antibiotics or hemodialysis. The only protective factor observed in the group of patients who received furosemide was a higher proportion of individuals vaccinated against COVID-19. Oximetry significantly improved during hospitalization in this group of patients, which might suggest that oxygenation is one of the clinical parameters through which furosemide had helped achieve higher survival with the drug, demonstrating a protective effect potentially through various mechanisms, mainly in less severe hospitalized patients.

Our stepwise multivariate statistical models were designed to identify factors associated with death in hospitalized COVID-19 patients, with a specific focus on evaluating the impact of furosemide use on mortality. The findings from our analysis revealed that certain clinical factors, such as having an elevated PSI score >120, requiring mechanical ventilation, and exhibiting specific hematologic markers, were identified as risk factors for mortality. Conversely, factors such as vaccination, furosemide use, and certain laboratory parameters were identified as independent protective factors against mortality. It is worth noting the significance of vaccination as a protective factor against mortality, emphasizing the crucial role of vaccination in reducing COVID-19-related deaths. Our findings on mortality risk shed light on the complex interplay of various factors contributing to COVID-19 mortality, including pre-existing conditions, disease severity, and the types of medical interventions received during the hospitalization stay. This intricate relationship has been extensively documented in the literature, as reported by numerous authors across different regions and published in systematic reviews and meta-analyses [1,5,27].

Our approach of stratifying patients based on clinical variables like disease severity and comorbidities enriches our analysis by providing a more detailed understanding of how furosemide’s impact on mortality risk varies across patient profiles. This approach allows us to discern patterns and nuances that might not be apparent when examining the entire patient cohort as a single group. Our early results revealed that the effect of furosemide on mortality risk differed across various strata defined by a combination of clinical variables, as outlined in Table 3. For instance, we observed a protective effect of furosemide against death in specific patient strata such as non-smokers, those not undergoing hemodialysis, and those without suspected bacterial infection. Moreover, furosemide also exhibited a protective effect among patients with improved clinical markers—such as lower PSI scores and reduced inflammation—as indicated by lower NLR levels. Interestingly, furosemide demonstrated a more pronounced protective effect in specific subgroups, particularly in unvaccinated patients and those requiring mechanical ventilation. However, it is essential to recognize that the overall impact of furosemide on modifying the risk of death was not substantial across most patient strata. This nuanced understanding underscores the complexity of furosemide’s effect on mortality and highlights the importance of considering patient-specific factors when evaluating its therapeutic potential in COVID-19 management.

Moreover, we observed that the effect of furosemide on death risk significantly changed based on the presence or absence of a PSI score >120 and hemodialysis requirement (stratum-specific effect modification). Furosemide administration significantly reduced death probability in patients with lower disease severity (PSI score <120 and non-hemodialysis). These results were confirmed by our interaction analysis (furosemide use *PSI score >120 *hemodialysis), which revealed a protective effect of furosemide on death risk in patients with lower disease severity compared to those with higher disease severity. In the group of patients with severe disease (PSI score >120 or requiring hemodialysis), furosemide use did not modify the risk of death or was associated with increased risk. The significant increase in survival time observed in patients receiving furosemide in this subgroup with lower disease severity underscores the potential therapeutic benefit of this medication in mitigating adverse outcomes associated with COVID-19 infection. However, the lack of a significant survival difference when considering all hospitalized patients suggests that the effect of furosemide may be specific to patient profiles.

The potential therapeutic benefit of furosemide in alleviating adverse outcomes associated with COVID-19 infection can be attributed to several mechanisms, primarily centered around its ability to improve respiratory function, fluid, and oxygenation status in patients [6,14,17,55,56,57]. Furosemide could exert its beneficial effects by acting as a diuretic to improve respiratory function in hospitalized COVID-19 patients by addressing fluid accumulation. Fluid buildup in the lungs, stemming from conditions like heart problems or inflammation, can compromise lung function and reduce oxygen levels. By facilitating the removal of excess fluid from the lungs, Furosemide helps to enhance lung function and oxygenation, thereby promoting better outcomes for patients battling COVID-19 [58]. Studies such as the one conducted by Mikami et al. [56] have reported an association between early furosemide administration and enhanced oxygenation in patients with acute heart failure. Our own study corroborates these findings, as evidenced by significantly higher oximetry levels observed in patients treated with furosemide, suggesting a potential mechanism contributing to improved survival rates. Additionally, other researchers have noted that furosemide’s effect on urine oxygenation is particularly evident in patients who demonstrate a positive response to the drug in terms of urinary flow [59]. 

Furthermore, furosemide has demonstrated efficacy in enhancing oxygenation across various disease states. For instance, in patients with acute heart failure, the early administration of furosemide (within 1 h of hospital arrival) resulted in a remarkable 16.7% improvement in oxygenation compared to patients who received it later in their treatment [56]. Similarly, in a study involving children with dengue fever, furosemide was found to significantly enhance parameters such as PO2, PaO_2_/FiO_2_, and A-a gradient [57]. Russotto et al. [60] suggested that in patients with acute respiratory distress syndrome (ARDS), factors beyond ventilatory support can influence their ability to breathe effectively. Specifically, the intensity of their breathing effort may be impacted by various stimuli unrelated to mechanical ventilation. For instance, the extent of lung inflammation can affect their respiratory drive by activating specific nerve pathways, such as pulmonary C-fibers and the vagus nerve [61], and the inhibition of pulmonary stretch receptors can also influence their breathing patterns [61]. Our findings highlight the complex interplay of physiological factors involved in respiratory function in ARDS patients, emphasizing the need for comprehensive management strategies [43,44,45] and underscoring the importance of further investigation into the role of furosemide in enhancing oxygenation and its impact on survival outcomes. 

Considering our results and previous reports on furosemide’s effects on pulmonary function and respiratory performance, we speculate that the effects of furosemide observed in our analysis could result from a combination of improved lung compliance, its anti-inflammatory properties [62], reduction of noncardiogenic lung edema, and modulation of pulmonary stretch receptor activity (which reduces dyspnea, although this is also related to compliance) [62]. Although more studies are necessary, the usefulness of furosemide in acute lung processes has been confirmed by others [56,63], although its effect is more significant if given early or when the kidney function is better. 

In addition, the medical literature also suggests that furosemide has multiple potential benefits for various disease states, not limited to patients with COVID-19 infections. Although furosemide is not a bronchodilator, it has been demonstrated to limit or prevent bronchospasm through a handful of mechanisms that are not fully understood, including the modulation of inflammatory cells and prostaglandin activity [64]. Similarly, in chronic obstructive pulmonary disease patients, inhaled furosemide has been shown to alleviate symptoms and improve pulmonary function testing significantly [65]. Inhaled furosemide has been postulated to alleviate breathlessness during exercise testing by regulating vagal afferent activity to the pulmonary stretch receptors [66]. Thus, the mechanism of furosemide is complex, and its inhibition of cytokines such as interleukin-6 and tumor necrosis factor-alpha can directly impact the inflammatory cascade involved in acute COVID-19 infections [13]. Moreover, a handful of studies have demonstrated furosemide’s antiviral properties, which are not limited to COVID-19 infections. Topical furosemide can reduce human papillomavirus load when applied to patients by blocking the potassium influx that DNA-based viruses require for replication [67]. Through this mechanism of Na^+^/K^+^/2Cl^−^ cotransporter inhibition, furosemide has shown the potential to curb the cytopathic effects of the human immunodeficiency virus [68]. 

This study sheds light on the impact of furosemide administration in hospitalized patients with COVID-19, particularly before the kidney function worsens. Our findings indicate that furosemide use is associated with improved survival, especially when administered before a significant elevation in urea or creatinine levels happens, which underscores the importance of early intervention with furosemide in managing fluid accumulation and renal dysfunction in COVID-19 patients, potentially contributing to better clinical outcomes. However, an improvement in oxygenation levels speaks in favor of furosemide beyond fluid status, as well as an improvement in pulmonary function and anti-inflammatory effect. Although our markers of systemic inflammation (CRP and NLR) were not significant in our models, we cannot rule out a local anti-inflammation effect mediated by furosemide and even an antiviral effect, both reported in the literature [15,67].

These insights contribute to our understanding of the complex dynamics underlying COVID-19 mortality and highlight avenues for further research and clinical management strategies to reduce mortality rates in hospitalized patients with COVID-19 in future epidemics when other therapies are not available, mainly in those patients living in areas with low resources and barriers to novel therapies. Further research is warranted to validate these findings and explore furosemide’s optimal timing and dosing in this patient population. Additionally, considering the multifactorial nature of COVID-19 outcomes, future studies should investigate the interplay between furosemide use and other clinical variables to better elucidate its therapeutic potential in improving patient outcomes.

Our study demonstrated several strengths. Firstly, we conducted association analyses while adjusting for important factors such as vaccination history, comorbidities, disease severity, and therapeutic strategies. Additionally, we performed analyses with repeated measurements of these clinical factors throughout hospitalization, providing a more thorough understanding of their impact over time. Moreover, we examined the effect of furosemide administration from various perspectives, including a multivariate association analysis, an assessment of mortality rates, and an analysis of survival curves. By adopting this comprehensive approach to analysis, our study’s conclusions are supported by a robust and detailed assessment of the data.

The findings from our study underscore the therapeutic potential of furosemide and related molecules in the management of severe COVID-19, emphasizing how specific structural characteristics influence drug efficacy. The interactions of sulfonamide groups in these compounds with key cytokine sites, particularly TNF-α and IL-6, suggest significant roles in modulating immune responses. Additionally, their effective binding to human albumin points to improved pharmacokinetic profiles crucial for clinical success. The effectiveness of ebselen and montelukast’s interaction with SARS-CoV-2 Mpro sites, as found in the existing literature [36,37], underscores the critical role of molecular structure in the design of COVID-19 treatments. This knowledge opens the door to new therapeutic strategies. While montelukast’s effects on specific molecular targets are well documented, our focus shifts to furosemide due to its unique chemical and physicochemical characteristics described in the literature [12,15,57,64]. Furosemide’s lower ClogP value not only indicates its suitability for various administration routes, including nebulized and systemic, but also highlights its potential for direct and indirect interactions with viral components. Studying furosemide and structurally similar molecules could lead to the discovery of new treatments and therapies for COVID-19. It is crucial to delve deeper into how these structures can be utilized effectively in the fight against the virus, emphasizing the need for comprehensive investigations into their mechanisms and therapeutic potential.

Despite these strengths, our study also had limitations. While the analysis to assess the potential of furosemide in reducing mortality among less severe patients (PSI < 120) without hemodialysis achieved a statistical power of 87.4% at an alpha level of 0.05, the limited number of patients who received furosemide suggests that a larger sample size would be desirable to observe differences between groups more accurately. Additionally, we did not consider the doses of furosemide administered, emphasizing the need for future studies to address this aspect. A notable aspect of the analyzed cohort was its high mortality rate of 43.9%. This aligns with hospital mortality reports in Mexico, which recorded a rate of 45.1% (95% CI 44.9–45.3) from March 2020 to August 2022, positioning Mexico among the countries with the highest hospital mortality globally during that period [3]; therefore, it is crucial to consider this when making comparisons with future studies. From a broader perspective, another critical aspect for discussion is the extensively documented long-term impact of COVID-19 on the respiratory, cardiovascular, and nervous systems. Long COVID has emerged as a significant global health concern due to its complex effects, including the dysregulation of the renin–angiotensin–aldosterone system (RAAS), inflammation, and coagulation issues [69]. The urgency and significance of thorough research into these enduring effects are essential for advancing strategies in prevention, rehabilitation, and overall patient care, highlighting the potential of furosemide in managing both acute infection and chronic respiratory complications.

One aspect that was not routinely obtained in hospitalized patients was their echocardiographic parameters of heart function, as well as data on ischemia or cardiac arrhythmias and their implications for patient outcomes. All patients had different systemic impairments that affected cardiac function in various ways, requiring multi-organ support. A very detailed study of cardiac functions was not routine in various guidelines during the early stages of the pandemic, and consensus guidelines for diagnosing and managing cardiac alterations in critically ill COVID-19 patients emerged when this project was in its final stages or thereafter [70,71,72]. Therefore, a future study with more detailed data on cardiac abnormalities and the impact of administered medications, including furosemide, is desirable. Additionally, future research involving patients who survived hospitalization due to COVID-19 would be of interest, where more detailed cardiological, pulmonary, and neurological analyses could be conducted, given the reported long-term effects [69,72].

## 5. Conclusions

Our study on hospitalized COVID-19 patients in Mexico indicates that furosemide, typically used as a diuretic, may have beneficial effects on survival, particularly in less severe cases. These effects are likely related not only to its conventional use for fluid management but also potentially to its anti-inflammatory and antiviral properties. Given the complex interplay of factors affecting COVID-19 outcomes, our findings suggest that the early and strategic use of furosemide could improve patient prognosis, especially in settings with limited access to advanced treatments. Further research is needed to confirm these results and refine treatment protocols, enhancing the management of COVID-19 and potentially other respiratory infections in diverse healthcare settings.

## Figures and Tables

**Table 1:** Main clinical characteristics of the participating subjects at the time of enrollment and prescribed treatments or drugs.

Clinical		Furosemide Use		
Characteristic	All	No	Yes	*p*-Value
	*n* = 515 (100%)	*n* = 439 (100%)	*n* = 76 (100%)	
≥60 years	63.0%	62.8%	63.9%	0.896
Male (%)	61.9%	60.9%	65.3%	0.515
Diabetes	43.3%	39.2%	66.7%	<0.001 *
HBP	42.3%	35.8%	79.2%	<0.001 *
BMI	30.3 ± 6.9	30.2 ± 7.2	31.6 ± 5.6	0.412
Smoking	7.6%	7.7%	7.5%	0.999 *
CKD	22.4%	16.1%	61.1%	<0.001
Heart disease **	3.4%	3.2%	4.2%	0.721
Charlson Index	3.6 ± 2.1	3.5 ± 2.1	4.5 ± 1.8	<0.001 *
Vaccine (any doses)	43.9%	41.5%	55.6%	0.029
Vaccine complete	37.3%	35.1%	50.8%	0.019
Clinical data upon hospital admission				
PSI	105 ± 40	104 ± 41	117 ± 28	0.048
Disease phase				0.006
Viral	38.1%	36.1%	50.0%	
Pulmonary	46.2%	49.0%	29.2%	
Inflammation	15.8%	14.9%	20.8%	
Oximetry	91.0 ± 11.1	91.1 ± 10.9	90.8 ± 12.1	0.855
Neutrophils	8503 ± 6234	7956 ± 5793	11,809 ± 7788	<0.001
Lymphocytes	923 ± 798	931 ± 837	880 ± 558	0.623
PlateletsX1000	257 ± 120	255 ± 114	272 ± 150	0.266
NLR	13.3 ± 14.1	12.6 ± 12.4	17.9 ± 21.4	0.004
D-Dimer	1639 ± 3163	1581 ± 3112	2230 ± 3707	0.430
ESR	29.5 ± 12.1	28.9 ± 11.7	32.7 ± 14.5	0.214
CRP	16.9 ± 19.6	17.1 ± 20.5	16.1 ± 11.6	0.755 *
Ferritin	737.7 ± 578.5	733.5 ± 542.6	807.5 ± 796.0	0.017
Creatinine	2.5 ± 3.7	2.0 ± 2.9	5.6 ± 6.1	<0.001
Urea	80.1 ± 67.2	70.3 + 58.1	137.4 + 87.4	<0.001
eGFR	65.8 ± 46.3	72.0 ± 46.4	30.2 ± 27.2	<0.001
AST	62.2 ± 183.5	55.8 ± 129.2	106.7 ± 388.6	0.075
ALT	47.4 ± 101.1	43.4 ± 69.0	74.6 + 218.2	0.047
ALP	105.4 ± 90.7	103.2 ± 95.0	127.5 ± 59.2	0.189
LDH	392.9 ± 414.8	385.8 ± 396.5	446.8 ± 530.6	0.294
Glucose	195.7 ± 144.9	191.1 ± 141.7	217.5 ± 165.1	0.176
INR	1.14 ± 0.32	1.14 ± 0.34	1.12 ± 0.15	0.715
Main prescribed treatments during hospitalization				
Anticoagulants	90.5%	89.9%	93.1%	0.520
Antibiotics	48.4%	46.3%	58.6%	0.038
Amine support	8.6%	8.5%	9.7%	0.658
Steroids	92.9%	91.0%	97.2%	0.990
Mech. Vent.	31.9%	31.7%	33.3%	0.786
Hemodialysis	10.3%	6.0%	34.7%	<0.001

* To compare groups (intergroup analysis), an unpaired Student’s *t*-test or Fisher’s exact test was used, as appropriate for numerical or qualitative data, respectively. BMI: body mass index. HBP: systemic arterial hypertension. Smoking: current smoker. ** Heart disease: history of ischemic disease or heart failure. CKD: chronic kidney disease. Complete vaccination regimen: at least 2 doses of ChAdOx1-S (AstraZeneca) or BNT162b2 (Pfizer). PSI: Pneumonia Severity Index. Neutrophils, lymphocytes, and plateletsX1000 are expressed in cells per microliter of blood. NLR: neutrophil/lymphocyte ratio: D-Dimer expressed in ng/mL. ESR: Erythrocyte Sedimentation Rate, in mm/hour. CRP: C-Reactive Protein, in mg/dL. Ferritin expressed in ng/mL. Creatinine expressed in mg/dL. eGFR: estimated glomerular filtration rate, expressed in mL/min/1.73 m^2^. ALP: alkaline phosphatase, expressed in international units per liter (IU/L). AST: aspartate aminotransferase, expressed in IU/L. ALT: alanine aminotransferase, expressed in IU/L. LDH: Lactate Dehydrogenase, expressed in IU/L. Glucose expressed in mg/dL. INR: prothrombin time expressed as an international normalized ratio. Mech. Vent.: mechanical ventilation. Oximetry: arterial oxygen saturation (Sao2) measured by arterial blood gas.

**Table 2:** Relative risk from multivariate generalized linear mixed models with binary logistic regression link of various clinical characteristics and vaccination schedules to have a fatal outcome in patients hospitalized for COVID-19.

	Model 1				Model 2			
		95% CI				95% CI		
Covariate	AdRR	Lower	Upper	*p*	AdRR	Lower	Upper	*p*
Diabetes	0.93	0.63	1.39	0.738				
HBP	0.95	0.65	1.39	0.779				
Smoking	3.89	2.19	6.91	<0.001	2.48	1.53	4.01	<0.001
Charlson Ind	7.37	2.03	26.79	0.002	6.86	2.46	19.11	<0.001
Vaccine	0.58	0.38	0.88	0.010	0.58	0.42	0.80	0.001
CKD	1.67	0.82	3.40	0.159				
Phase	1.94	1.32	2.85	0.001	1.92	1.41	2.61	<0.001
PSI ≥ 120	7.11	4.92	10.27	<0.001	7.89	5.82	10.70	<0.001
Neutrophilia	2.19	1.46	3.29	<0.001	2.12	1.52	2.95	<0.001
NLR	2.06	1.36	3.11	0.001	2.39	1.72	3.32	<0.001
Ferritin	0.90	0.63	1.29	0.574				
ALT ≥ 45 UL	0.60	0.39	0.92	0.018	0.71	0.50	1.00	0.049
eGFR ≥ 60	0.67	0.37	1.20	0.179				
Creatinine	0.57	0.21	1.51	0.255				
Urea > 135	1.38	0.63	3.02	0.421				
Mech. Vent.	23.59	16.46	33.80	<0.001	23.12	17.28	30.92	<0.001
Hemodialysis	1.75	0.86	3.59	0.125	1.85	1.07	3.19	0.027
Antibiotic	1.71	1.19	2.45	0.004	1.52	1.13	2.06	0.006
Furosemide	0.58	0.36	0.92	0.022	0.60	0.41	0.87	0.008

AdRR: adjusted relative risk. The multivariate statistical model included all the listed characteristics (fixed effects) that were considered risk factors for mortality, with their absence classified as the reference. Adjustments were made for all variables listed in the model. The pandemic period time was included in the model as a random effect. Model 1 includes the variables that were different between patients who received furosemide during their hospitalization and those who did not (see Table 1), with the addition of variables such as mechanical ventilation and smoking considered relevant predictors of death. Model 2 only includes variables that, in the analysis of Model 1, resulted in a *p*-value of less than 0.150. HBP: systemic arterial hypertension. Smoking: current smoker. Charlson Ind: Charlson Index ≥ 8. CKD: chronic kidney disease. Phase: advanced disease phase (pulmonary/hyperinflammatory) upon admission (reference: viral phase). eGFR > 60: estimated glomerular filtration rate, expressed in mL/min/1.73 m^2^. PSI: Pneumonia Severity Index. Mech. Vent.: mechanical ventilation. Vaccine: at least 1 dose of any vaccine. Neutrophilia: >9.1 × 10^3^/µL. Ferritin: expressed in ng/mL. NLR: neutrophil-to-lymphocyte ratio > 14.6. Creatinine expressed in mg/dL. ALT: alanine aminotransferase, expressed in IU/L. Urea > 135 mg/dL. The dichotomization of variables was determined through an analysis of the area under the receiver operating characteristic (ROC) curve, with its respective cutoff point calculated to discriminate subjects who died.

**Table 3:** Stratum-specific furosemide effect on fatal outcome in different strata, according to the presence or absence of a clinical characteristic, in patients hospitalized for COVID-19.

Stratum-Specific		Effect of Furosemide			
	Present	AdRR	95% CI Lower–Upper		*p*
All patients		0.60	0.41	0.87	0.008
Smoking	No	0.55	0.37	0.82	0.003
	Yes	1.42	0.77	2.60	0.248
Charlson Index ≥ 8	No	0.65	0.43	0.97	0.037
	Yes	0.62	0.43	0.90	0.014
Vaccine	No	0.53	0.36	0.78	0.001
	Yes	0.87	0.39	1.90	0.718
Advanced phase	No	0.67	0.30	1.48	0.323
	Yes	0.70	0.48	1.14	0.151
PSI score ≥ 120	No	0.25	0.14	0.46	<0.001
	Yes	1.76	0.96	3.24	0.069
Neutro ≥ 9.1 × 10^3^/µL	No	0.54	0.33	0.90	0.019
	Yes	0.71	0.42	1.21	0.208
NLR > 14.6	No	0.45	0.27	0.75	0.002
	Yes	0.79	0.47	1.34	0.394
ALT ≥ 45 UL	No	0.68	0.44	1.06	0.092
	Yes	0.40	0.22	0.74	0.003
Mech. Vent.	No	1.03	0.52	2.04	0.092
	Yes	0.45	0.29	0.70	<0.001
Hemodialysis	No	0.45	0.30	0.68	<0.001
	Yes	2.30	0.73	7.23	0.153
Antibiotic	No	0.22	0.11	0.45	<0.001
	Yes	1.09	0.72	1.65	0.672

AdRR: adjusted relative risk. The multivariate statistical model included all the characteristics (fixed effects) listed in Table 3 that were considered risk factors for mortality, with their absence being classified as the reference, except for the type of vaccine administered, where the reference was the unvaccinated group. Adjustments were made for all variables listed. The pandemic period time was included in the model as a random effect. Smoking: current smoker. PSI: Pneumonia Severity Index. Vaccine: at least 2 doses of ChAdOx1-S or BNT162b2; 1 dose of Ad5-nCoV or CoronaVac was also considered, although these vaccines were poorly represented (less than 4% of patients between these two types of vaccines). Neutro ≥ 9.1 × 10^3^/µL: neutrophils. NLR: neutrophil-to-lymphocyte ratio > 14.6. ALT: alanine aminotransferase, expressed in IU/L. Mech. Vent.: mechanical ventilation. The dichotomization of variables was determined through an analysis of the area under the receiver operating characteristic (ROC) curve, with its respective cutoff point calculated to discriminate subjects who died.

**Table 4:** Relative risk of the model involving a 3-way interaction of the principal variables with stratum-specific furosemide effect modification on the risk of death in hospitalized patients with COVID-19.

Variables in Interaction				95% CI		
Furosemide	PSI ≥ 120	Hemodialysis	AdRR	Lower	Upper	*p*
No	No	No	1.000 ^a^			
Yes	No	No	0.227	0.149	0.345	<0.001
No	Yes	No	9.382	7.671	11.474	<0.001
Yes	Yes	No	9.326	5.571	15.613	<0.001
No	No	Yes	3.779	2.270	6.289	<0.001
Yes	No	Yes	4.576	1.944	10.774	0.001
No	Yes	Yes	5.742	4.023	8.197	<0.001
Yes	Yes	Yes	15.996	9.775	26.177	<0.001

AdRR: adjusted relative risk. The multivariate statistical model included all the following characteristics (as fixed effects): Charlson Index ≥8, vaccination, and mechanical ventilation, in addition to 3-way interaction analyses (furosemide use *PSI score >120 *hemodialysis). The pandemic period time was included in the model as a random effect. ^a^ Value of contrast.

**Table 5:** Furosemide use in patients hospitalized for COVID-19, with a PSI score <120, and without hemodialysis treatment.

Furosemide		Death: *n* (%)		
Use	Total	No	Yes	*p* *
No	265 (100%)	197 (74.3%)	68 (25.7%)	0.008
Yes	27 (100%)	26 (96.3%)	1 (3.7%)	

* Fisher’s exact test.

**Table 6:** Clinical characteristics of hospitalized COVID-19 patients with a PSI score <120 and without hemodialysis treatment, according to the status of furosemide use, upon hospital admission and throughout hospitalization.

Clinical	Upon Hospital Admission			Throughout Hospitalization		
Characteristic	Furosemide			Furosemide		
	No	Yes	*p* *	No	Yes	*p* *
Male	58.3%	59.3%	0.548			
Age (years)	60.2 ± 16.4	63.9 ± 13.8	0.256	60.1 ± 15.8	63.7 ± 10.7	0.001
CKD	8.3%	40.7%	<0.001	NA **	NA **	
Vaccine	45.8%	54.2%	0.120	NA **	NA **	
Mech. Vent.	1.5%	3.7%	0.402	2.3%	11.1%	0.041
Antibiotic	4.9%	3.7%	0.780	42%	63%	0.043
PSI	81.5 ± 24.3	95.5 ± 16.2	0.004	80.4 ± 24.1	94.1 ± 16.0	<0.001
Oximetry	92.3 ± 10.5	93.5 ± 8.9	0.578	92.6 ± 8.3	95.6 ± 3.8	0.012
ESR	29.7 ± 10.4	36.5 ± 18.4	0.091	30.4 ± 10.7	28.5 ± 11.4	0.282
Neutrophils	6985 ± 4699	10,340 ± 7743	0.001	7353 ± 5025	8145 ± 5409	0.065
Lymphocytes	986 ± 875	854 ± 312	0.441	971 ± 830	970 ± 502	0.988
NLR	10.4 ± 9.0	13.9 ± 12.5	0.068	11.4 ± 15.0	10.0 ± 7.9	0.263
PlateletsX1000	255 ± 111	227 ± 140	0.219	302 ± 136	277 ± 186	0.045
PLR	817.5 ± 1530.9	272.8 ± 161.7	0.066	938.9 ± 1978.5	834.7 ± 2025.8	0.535
eGFR	84.7 ± 45.9	37.7 ± 28.1	<0.001	109.6 ± 60.5	66.0 ± 44.5	<0.001
Creatinine	1.5 ± 2.4	3.1 ± 3.1	<0.001	1.2 ± 2.3	2.3 ± 2.9	<0.001
Urea	52.0 ± 42.4	87.6 ± 44.9	<0.001	57.1 ± 42.3	85.1 ± 63.4	<0.001
AST	45.9 ± 58.8	59.8 ± 79.6	0.330	40.5 ± 51.1	45.7 ± 33.5	0.328
ALT	41.6 ± 40.9	40.5 ± 49.6	0.907	49.0 ± 56.8	41.2 ± 25.6	0.177
ALP	101.5 ± 100.8	153.7 ± 71.3	0.071	86.3 ± 55.0	196.6 ± 184.2	<0.001
LDH	366.1 ± 448.6	306.1 ± 129.9	0.542	341.3 ± 186.3	248.4 ± 116.5	<0.001
Glucose	172.1 ± 110.1	205.2 ± 107.0	0.145	149.0 ± 81.7	168.0 ± 102.6	0.009
D-Dimer	771.1 ± 1102.4	717.0 ± 314.3	0.866	1305.9 ± 2262.4	1747.2 + 1722.2	0.089
CRP	15.5 ± 20.1	16.5 ± 9.6	0.852	7.3 ± 7.9	8.8 ± 5.8	0.419
Ferritin	678.2 ± 506.6	477.8 ± 420.3	0.171	792.9 ± 597.6	920.0 ± 778.0	0.291

* To compare groups (intergroup analysis), an unpaired Student’s *t*-test or Fisher’s exact test was used, as appropriate for numerical or qualitative data, respectively. ** NA: not analyzed, since the value of this variable does not change during the patient’s hospitalization. Percentages or averages and standard deviations are shown. CKD: chronic kidney disease. PSI: Pneumonia Severity Index. Oximetry: arterial oxygen saturation (Sao2) measured by arterial blood gas. Neutrophils, lymphocytes, and plateletsX1000 are expressed in cells per microliter of blood. NLR: neutrophil/lymphocyte ratio. PLR: platelet/lymphocyte ratio. D-Dimer expressed in ng/mL. ESR: Erythrocyte Sedimentation Rate, in mm/hour. CRP: C-Reactive Protein, in mg/dL. Ferritin expressed in ng/mL. Creatinine expressed in mg/dL. eGFR: estimated glomerular filtration rate, expressed in mL/min/1.73 m^2^. ALP: alkaline phosphatase, expressed in international units per liter (IU/L). AST: aspartate aminotransferase, expressed in IU/L. ALT: alanine aminotransferase, expressed in IU/L. LDH: Lactate Dehydrogenase, expressed in IU/L. Glucose expressed in mg/dl. Mech. Vent.: mechanical ventilation.

**Table 7:** Cutoff scores, area under the curve (AUC), for various clinical measures examined in this study for predicting furosemide use in patients hospitalized for COVID-19.

Variable	Group	AUC	SE	95%CI		Cutoff	*p*	Difference *	
								AUC	*p*
PSI	Lived	0.587	0.034	0.520	0.655	90.5	0.011	0.082	0.039
	Died	0.506	0.019	0.468	0.544	141.5	0.766		
Oximetry	Lived	0.551	0.038	0.477	0.626	80.5	0.176	0.054	0.215
	Died	0.498	0.021	0.457	0.539	73.5	0.910		
Creatinine	Lived	0.730	0.033	0.666	0.794	2.6	<0.001	0.088	0.020
	Died	0.642	0.019	0.604	0.679	3.5	<0.001		
Urea	Lived	0.779	0.027	0.726	0.832	53.2	<0.001	0.164	<0.001
	Died	0.614	0.022	0.572	0.657	87.5	<0.001		
eGFR	Lived	0.301	0.035	0.233	0.369	84.7	<0.001	−0.047	0.229
	Died	0.348	0.019	0.312	0.385	53.7	<0.001		

A score equal to or higher than the cutoff point is the predictor of furosemide use. AUC: area under the curve. SE: standard error. eGFR: estimated glomerular filtration rate, expressed in mL/min/1.73 m^2^. PSI: Pneumonia Severity Index. Oximetry: arterial oxygen saturation (Sao2) measured by arterial blood gas. Creatinine expressed in mg/dL. Urea expressed in mg/dL. * Difference in AUC between patients who were discharged due to improvement (lived) or due to death.

## Data Availability

The original contributions presented in the study are included in the article; further inquiries can be directed to the corresponding author/s.
